# Supplementary material for: A multivariate analysis of CalEnviroScreen: comparing environmental and socioeconomic stressors versus chronic disease
Source: Environ Health. 2017 Dec 13;16:131. doi: 10.1186/s12940-017-0344-z (PMC5729424; doi:10.1186/s12940-017-0344-z)
Supplement: Supplementary file 1 — Supporting information (PDF, 11 pages). Includes the following: methods for spatial joining of CalEnviroScreen versus hospital data; correlation of variables in ICD-9 hospital data (text and Table S2); study variables description (Table S1); scatterplot matrix of hospital visit rate parameters (Figure S1); total hospital ICD-9 codes vs. total population (Figure S2); and scatterplot matrix of socioeconomic variables (Figure S3). (PDF 1699 kb) [file 12940_2017_344_MOESM1_ESM.pdf]

## Supporting Information

A multivariate analysis of CalEnviroScreen: comparing environmental and socioeconomic stressors versus chronic disease

Ben K. Greenfield [1] *, Jayant Rajan [2] and Thomas E. McKone [1]

1. Environmental Health Sciences Division, School of Public Health, University of California, Berkeley, 50 University Hall #7360, Berkeley, CA, 94720, USA.

2. Department of Medicine, Zuckerberg San Francisco General Hospital, University of California, San Francisco, 1001 Potrero Avenue, San Francisco, CA 94110, USA.

* Corresponding Author. Current Address: Environmental Sciences Department, Southern Illinois University – Edwardsville, IL 62026, USA.

email: begreen@siue.edu

**Spatial joining of CalEnviroScreen versus hospital data**

The spatial levels of organization for the CalEnviroScreen (CES) versus ICD-9 datasets are different, requiring generation of a geographic correspondence. While CES is organized at the census-tract scale, the spatial reference point for ICD-9 is the zip code. Census tracts are precise polygon features, whereas zip codes correspond to linear USPS route areas, post office box collection points, or large volume customer collection points. Therefore, zip codes require further treatment to allow correspondence with polygon features or area-based calculations [1]. Zip code tabulation areas (ZCTA) are polygons, developed and employed by the US Census to approximate the areal coverage of zip codes, and as such are appropriate for area-based calculations [2]. Because ZCTA is an aggregate scale that can be used to test for and address spatial autocorrelation, we incorporated all data up to the scale of ZCTA. However, ZCTAs are similar but not identical to zip codes, such that additional processing is needed to extrapolate between measures, and avoid data loss. The following procedure was employed to achieve these goals:

1. The ESRI 2014 zip code point data layer and the ESRI ZCTA boundary spatial polygon layer (based on 2010 Census) were downloaded from the web. Point estimates of zip codes were obtained from ESRI's zip code point file layer (http://www.arcgis.com/home/item.html?id=1eeaf4bb41314febb990e2e96f7178df) on June 14, 2015. ZCTA polygon estimates were downloaded on May 19, 2015.
2. ICD-9 zip codes that represent post office boxes or large volume customers were assigned to ZCTA in ArcMap by doing a spatial join (point to polygon) between the ESRI zip code point layer and the ESRI ZCTA polygon layer. That is, each ZCTA was defined to contain the corresponding zip code area, in addition to all PO boxes and large volume customers located within the geographic boundaries of that ZCTA. The ZCTA polygons were also automatically set to represent the spatial locations for all zip code areas (i.e., not PO boxes or large volume customers).
3. Each ZCTA has a total population estimate in the 2010 US Census. These data were downloaded from www.socialexplorer.com on May 13, 2015. This was used as the denominator term in the disease burden measure.
4. ICD-9 diagnosis frequencies were summed from all corresponding zip codes by ZCTA. That is, diagnosis counts from all zip codes including PO Box and zip code areas were summed within the corresponding ZCTA.
5. The ICD-9-based disease burden measure was determined for each ZCTA (see main text Methods for details)
6. CalEnviroScreen (CES) results and the derived multivariate indicators (i.e., PCA results) were aggregated from the census-tract scale to the ZCTA scale using the area-weighted and population-weighted average of corresponding values. Data aggregation was performed on the census tracts and parameters from the original CES October, 2014 dataset after transformation, and also on the PCA results. The population-weighting scheme was based on results from the MABLE/Geocorr12 population weighting algorithm [3], which were downloaded on September 30, 2015.

7. Simultaneously autoregressive models (SAR) require all polygons to have adjacent neighbors; there can be no isolated polygons. Therefore, geographically isolated ZCTAs having CES data needed to be removed from the analysis. Four isolated ZCTAs were removed: 93608 (Cantua Creek), 95257 (Wilseyville), 95925 (Challenge), and 96052 (Lewiston). All of these sites were in rural areas, with low to moderate CES scores (8.4 to 41.9), a low overall population (152 to 2196 residents in the 2010 census), and mostly white (Caucasian) inhabitants (51% to 90%). Two ZCTAs were separated from other ZCTAs by narrow channels: 94501 (city of Alameda) and 92662 (Newport Beach). These two ZCTAs were manually joined to adjacent ZCTA polygons using the ArcMap editor.
8. Linear model and SAR model analyses were performed on the resulting data at the ZCTA scale.

**Correlation among diseases in ICD-9 database**

In the study analyses, we opted to focus on hospitalization rate for a subset of specific diagnoses, rather than the total frequency of ICD-9 diagnoses, as the measure of disease burden (see Methods). The multivariate correlation structure for reported ICD-9 codes (count $person^{-1}$ $yr^{-1}$) was examined across all zip codes (N = 1667) for each of the individual diagnoses, as well as the total rate of hospitalizations with at least one of the diagnoses (i.e., the “disease burden measure” in the main text). These data were square root transformed to achieve multivariate normality and analyzed via Pearson’s correlation coefficients (r). A positive correlation was observed for almost all pairwise comparisons (Table S2). Correlations were strong among total diagnosis rate, pneumonia, chronic obstructive pulmonary disease (COPD), asthma, myocardial infarction (MI; heart attack), and cerebral vascular accident (CVA; stroke) (Table S2, Fig. S1). In a principal component analysis of this correlation matrix, the first principal component (PC), which was negatively associated with all diseases, explained 45% of the variance in the data set. This PC was also very strongly associated with total reported codes (Pearson’s r = −0.90). These findings indicate that examination of the separate diagnoses would only provide limited unique information. This supported the development of a general indicator of disease burden that combines the different results. For this reason, we employed the disease burden measure, as described in the text.

**Table S1.** Parameters used in analysis, description, original units, and transformation applied. All environmental and socioeconomic parameters were developed at the census-tract scale and are summarized here, following OEHHA [4] and Faust et al. [5]. Hospital use was available at the zip code scale.

| Name | Parameter Class | Description | Units | Years | Transformation |
|---|---|---|---|---|---|
| Ozone | Environmental | Average daily maximum concentration | ppm | 2011-2013 | Cube root |
| $PM_{2.5}$ | Environmental | Annual mean concentration | $\mu g\ m^{-3}$ | 2011-2013 | Untransformed |
| Diesel PM | Environmental | Estimated daily emissions in a 4 $km^2$ area for a July workday from combined on-road and off-road sources | $kg\ d^{-1}$ | 2012 | $Log_{10}$ |
| Traffic density | Environmental | Sum of traffic volume (vehicle km) per unit time per road length within 150 m buffer of census tract border | Vehicle $km\ h^{-1}\ km^{-1}$ | 2013 | $Log_{10}$ |
| Drinking water | Environmental | Drinking water contaminant metric, defined as the sum of percentiles for concentration of 13 water contaminants and two violations (Maximum Contaminant Level and Total Coliform Rule) | % | 2005, 2009, 2013 | Arcsine (square root) |
| Pesticides | Environmental | Pesticide active ingredient used in production agricultural applications per unit area; includes 70 hazardous and volatile pesticides | $lb\ mi^{-1}$ | 2012-2014 | $Log_{10}$[a] |
| Toxic release | Environmental | Modeled toxicity-weighted chemical concentrations released to air from industrial facilities; data were obtained from USEPA Toxic Release Inventory, following the Risk Screening Environmental Indicators tool. | $lb\ yr^{-1}$ | 2011-2013 | $Log_{10}$ |
| Water body impairments | Environmental | Total number of pollutants listed as impairments in 303d Total Maximum Daily Load regulatory program | Unitless | 2012 | Cube root |
| Groundwater threats | Environmental | Weighted [b] sum of sites that could adversely impact groundwater, such as leaking underground chemical storage tanks, dry cleaners, dairies, airports, and produced-water ponds from oil and gas well stimulation. Sites were obtained from the GeoTracker data base maintained by the CA State Water Resources Control Board. | Unitless | 2016 | $Log_{10}$[a] |

| | | | | | |
|---|---|---|---|---|---|
| Cleanup sites | Environmental | Weighted [b] sum of sites with cleanup planned or underway due to hazardous chemicals. Sites were obtained from the EnviroStor data base maintained by the CA Department of Toxic Substances Control. | Unitless | 2016 | Cube root |
| Hazardous waste sites | Environmental | Weighted [b] sum of hazardous waste permitted facilities and sites that generate hazardous waste. Sites were obtained from the EnviroStor hazardous waste facilities data base maintained by the CA Department of Toxic Substances Control. | Unitless | 2016 | $Log_{10}$ [a] |
| Solid waste sites | Environmental | Weighted [b] sum of sites or facilities containing solid waste. Site types include waste disposal and composting sites, waste tire sites, scrap metal recyclers, or other site types that may contain and release hazardous materials | Unitless | 2016 | $Log_{10}$ [a] |
| Education | Socioeconomic | Percent of population >25 years old without a high school degree or equivalent | % | 2011-2015 | Cube root |
| Linguistic isolation | Socioeconomic | Percent of households defined by US Census as “limited English-speaking” | % | 2011-2015 | Cube root |
| Poverty | Socioeconomic | Percent of population with household income less than two times the federal poverty line | % | 2011-2015 | Square root |
| Unemployment | Socioeconomic | Percent of population >16 years old and workforce eligible that is unemployed | % | 2011-2015 | Cube root |
| Housing burden | Socioeconomic | Percent of households that is low income and paying >50% of income for housing costs | % | 2009-2013 | Square root |
| Over 65 | Demographic | Percent of population ≥ 65 years old (US Census) | % | 2010 | Square root |
| Asthma | Health outcome | Rate of emergency room visits for asthma (age adjusted) | Visits /10,000 people | 2011-2013 | Square root |
| Cardiovascular disease | Health outcome | Rate of emergency room visits for acute myocardial infarction (age adjusted) | Visits /10,000 people | 2011-2013 | Square root |
| Low birth weight | Health outcome | Percent of live singleton births weighing < 2.5 kg | % | 2006-2012 | Untransformed |
| Disease burden measure (DB) | Health outcome | Total number of instances of ICD-9 code reporting events per year per total population within zip code tabulation area | Events $person^{-1}$ $yr^{-1}$ | 2008 - 2011 | $\text{Sign}(\sqrt{\text{DB}}) * \ln(\lvert\text{DB}\rvert + 1)$ [c] |

a. To account for the presence of zeros, values were $\log_{10}$ transformed following the order-of-magnitude stabilizing procedure described in McCune and Grace [6]: $y = \log_{10}(x + 10^{C}) - C$, where $C = \text{floor}(\log_{10}(\min(x)))$

b. Site weighting was based on expected hazard of the site type and distance from populated census block within the census tract [5]

c. Modulus transformation per John and Draper [7]

**Table S2.** Pearson's correlation coefficients among the prevalence of ICD-9 codes for 14 specific disease diagnoses, examined in California Zip code tabulation areas. Had Dx: indicates the presence of any of the 14 diagnoses for a specific visit. The color of the boxes indicates strength and direction of the association, with red indicating negative association and blue indicating positive association.

| | Had Dx | Pneumonia | COPD | Asthma | MI | CVA | Diarrhea | Pancreatic cancer | Lung cancer | Breast cancer | Lymphoma | Leukemia | Depression | Schizophrenia |
|---|---|---|---|---|---|---|---|---|---|---|---|---|---|---|
| Pneumonia | 0.87 | | | | | | | | | | | | | |
| COPD | 0.89 | 0.85 | | | | | | | | | | | | |
| Asthma | 0.77 | 0.68 | 0.60 | | | | | | | | | | | |
| MI | 0.73 | 0.69 | 0.65 | 0.58 | | | | | | | | | | |
| CVA | 0.77 | 0.72 | 0.70 | 0.61 | 0.73 | | | | | | | | | |
| Diarrhea | 0.16 | 0.13 | 0.06 | 0.17 | 0.15 | 0.13 | | | | | | | | |
| Pancreatic cancer | 0.29 | 0.24 | 0.18 | 0.22 | 0.33 | 0.31 | 0.21 | | | | | | | |
| Lung cancer | 0.54 | 0.49 | 0.54 | 0.41 | 0.41 | 0.50 | 0.07 | 0.24 | | | | | | |
| Breast cancer | 0.38 | 0.30 | 0.28 | 0.32 | 0.33 | 0.42 | 0.18 | 0.35 | 0.32 | | | | | |
| Lymphoma | 0.54 | 0.47 | 0.38 | 0.51 | 0.49 | 0.49 | 0.24 | 0.36 | 0.33 | 0.44 | | | | |
| Leukemia | 0.31 | 0.28 | 0.22 | 0.27 | 0.31 | 0.33 | 0.19 | 0.28 | 0.24 | 0.27 | 0.31 | | | |
| Depression | 0.42 | 0.29 | 0.19 | 0.43 | 0.38 | 0.37 | 0.30 | 0.29 | 0.21 | 0.38 | 0.46 | 0.29 | | |
| Schizophrenia | 0.53 | 0.39 | 0.36 | 0.47 | 0.29 | 0.28 | 0.28 | 0.23 | 0.16 | 0.21 | 0.38 | 0.18 | 0.51 | |
| Low birth weight | 0.04 | 0.07 | −0.09 | 0.17 | 0.02 | 0.02 | 0.13 | 0.12 | −0.02 | 0.08 | 0.14 | 0.10 | 0.11 | 0.21 |

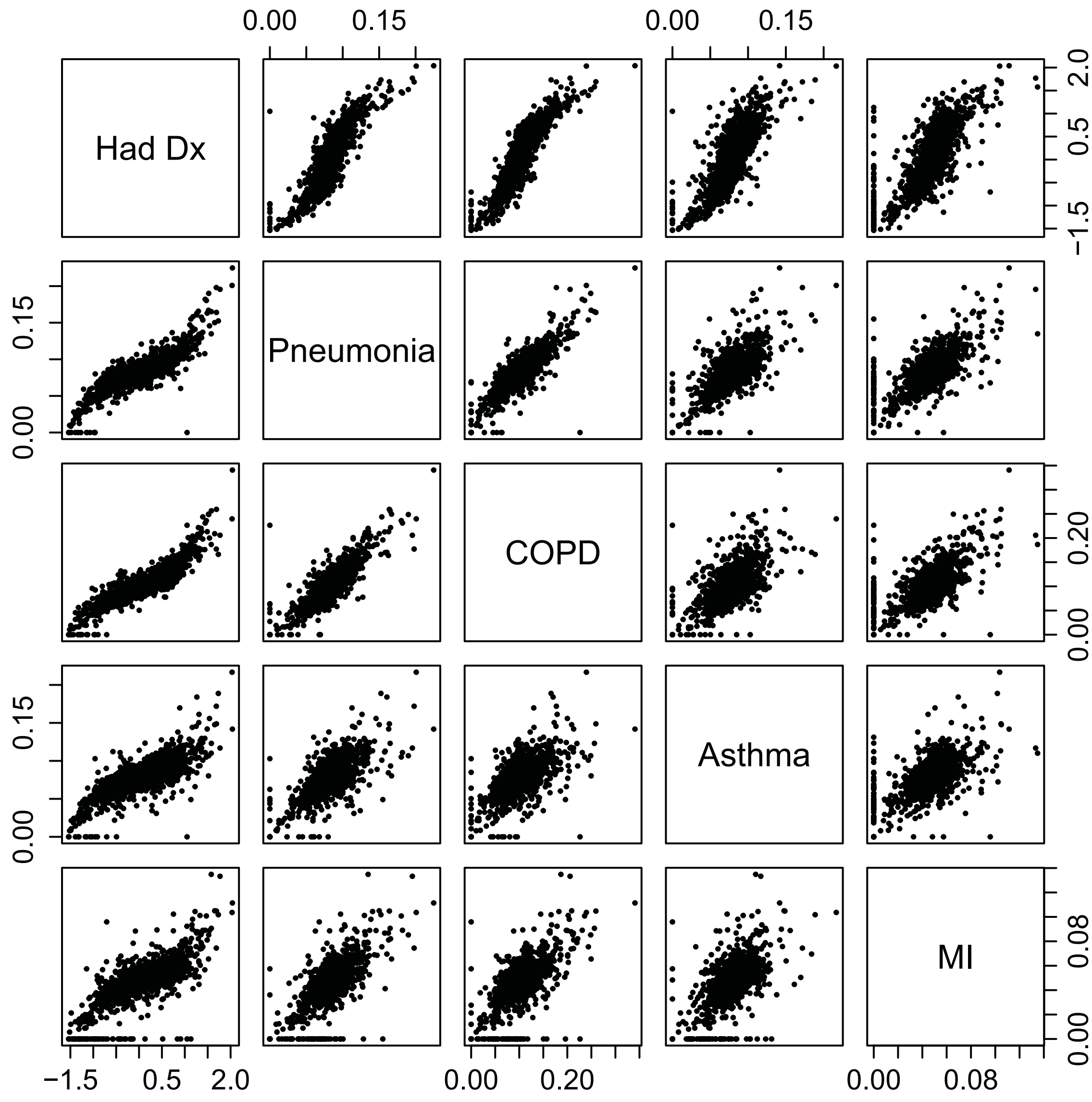


**Fig. S1.** Scatterplot matrix of selected hospital visit rate (health outcome) parameters. All data were normalized to ZCTA total population and square root transformed. Had Dx: indicates the total rate of hospitalization for at least one of the diagnoses; COPD = chronic obstructive pulmonary disease; MI = myocardial infarction (heart attack).

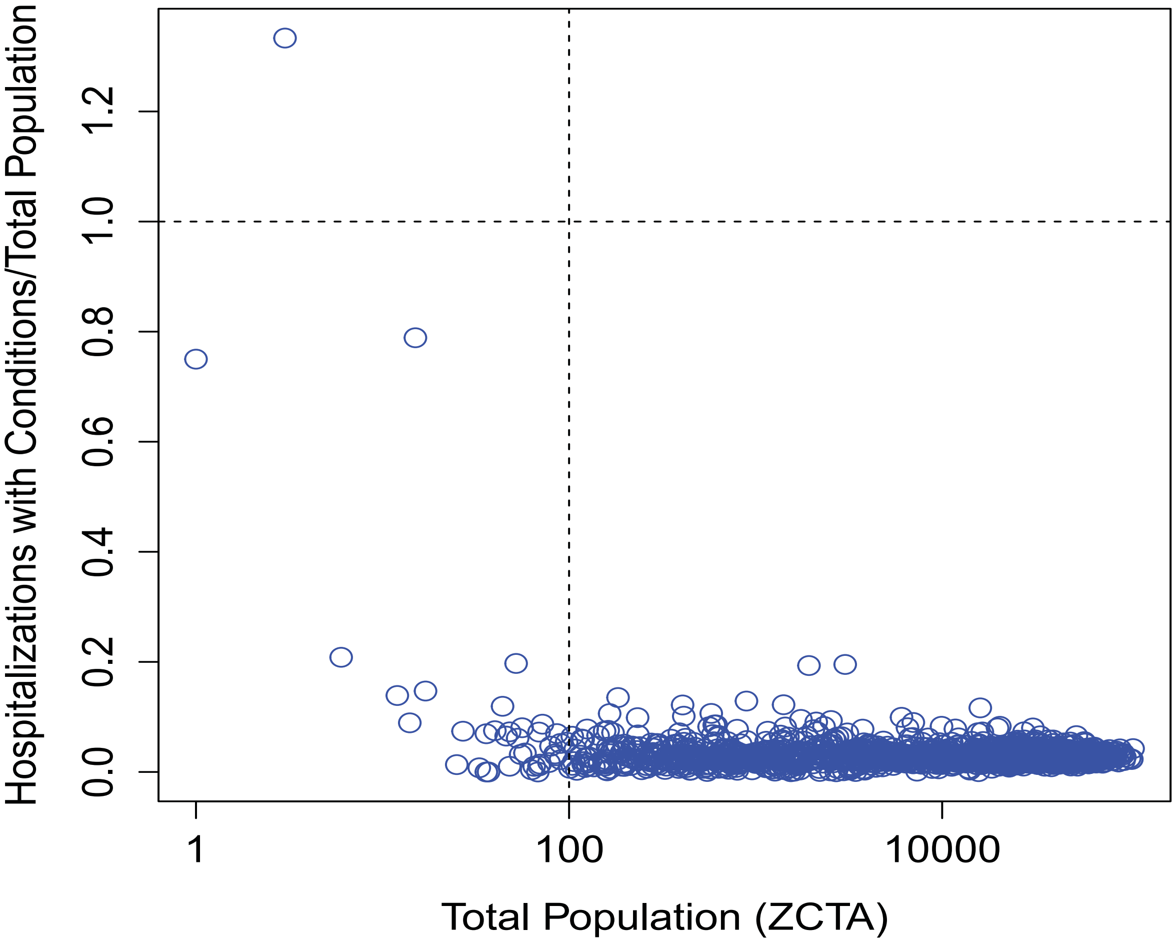


**Fig. S2.** Total hospital ICD-9 diagnostic codes (count person$^{-1}$ yr$^{-1}$) versus total population in each ZCTA (zip code tabulation area). Vertical dotted line: total population of 100; Horizontal dotted line: diagnostic code count of 1. The plot illustrates that for ZCTA with populations below 100, variability increased substantially. These ZCTA were thus removed from further analysis.

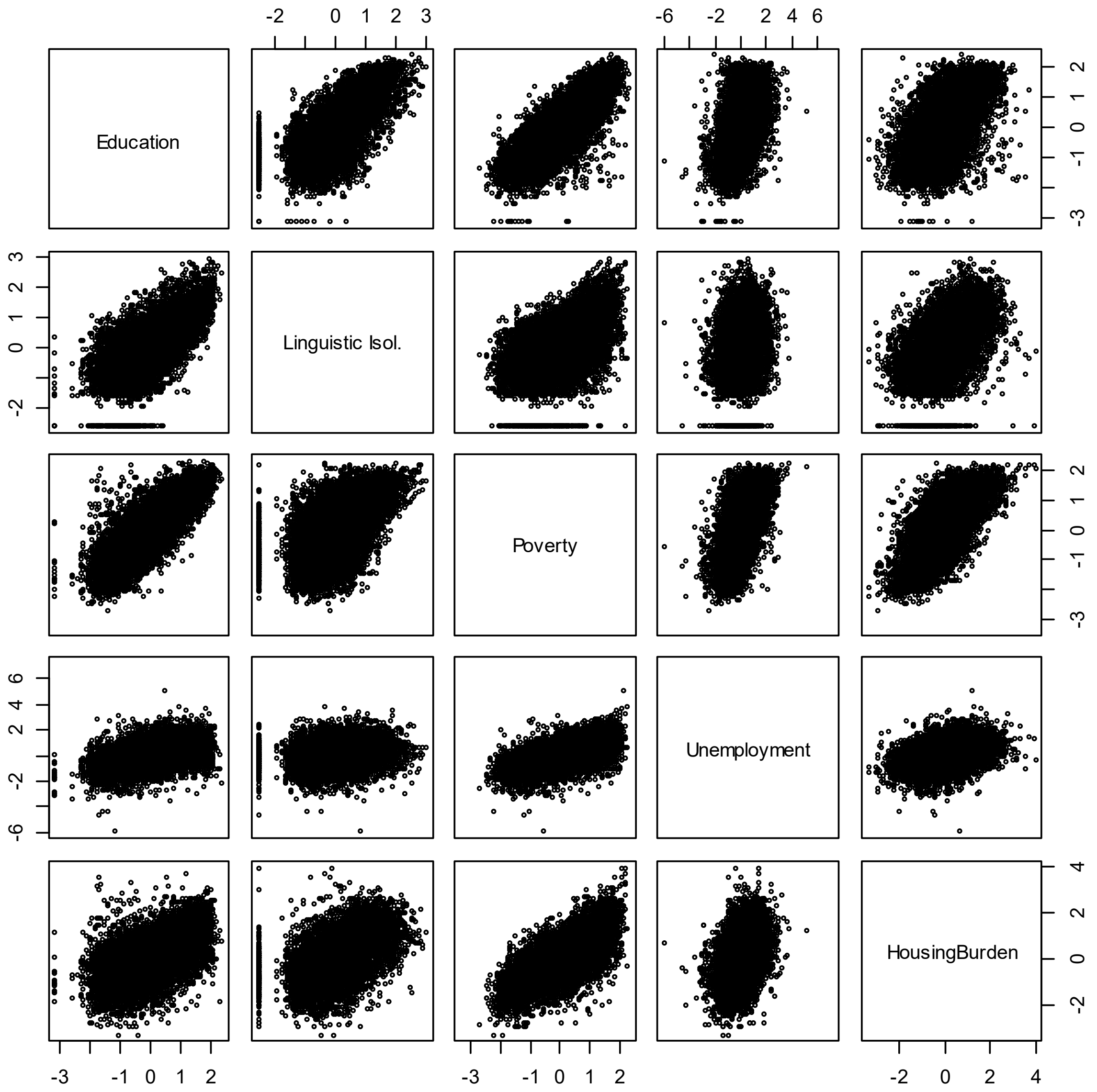


**Fig. S3.** Scatterplot matrix indicates positive correlation among the socioeconomic vulnerability indicators.

**Supporting Information References**

1. Grubesic TH, Matisziw TC. On the use of ZIP codes and ZIP code tabulation areas (ZCTAs) for the spatial analysis of epidemiological data. Int. J. Health Geogr. 2006;5:58.

2. U.S. Census Bureau. ZIP Code™ Tabulation Areas (ZCTAs™) [Internet]. 2015 [cited 2015 Sep 29]. Available from: https://www.census.gov/geo/reference/zctas.html

3. Missouri Census Data Center. MABLE/Geocorr12: Geographic Correspondence Engine [Internet]. 2012 [cited 2015 Jun 17]. Available from: http://mcdc.missouri.edu/websas/geocorr12.html

4. OEHHA. New in CalEnviroScreen 3.0: changes since version 2.0. Sacramento, CA; 2017.

5. Faust J, August L, Bangia K, Galaviz V, Leichty J, Prasad S, et al. Update to the California Communities Environmental Health Screening Tool, CalEnviroScreen 3.0 [Internet]. 2017. Available from: https://oehha.ca.gov/calenviroscreen/report/calenviroscreen-30

6. McCune B, Grace JB. Analysis of Ecological Communities [Internet]. Gleneden Beach, OR: MJM Software Design; 2002. Available from: www.pcord.com

7. John JA, Draper NR. An alternative family of transformations. J. R. Stat. Soc. Ser. C Appl. Stat. [Internet]. 1980;29:190–7. Available from: http://www.jstor.org/stable/2986305
